# Response Strategies of Root System Architecture to Soil Environment: A Case Study of Single-Species *Cupressus funebris* Plantations

**DOI:** 10.3389/fpls.2022.822223

**Published:** 2022-04-14

**Authors:** Wenchun He, Chao Luo, Yang Wang, Xiaochen Wen, Yu Wang, Tianyi Li, Gang Chen, Kuangji Zhao, Xianwei Li, Chuan Fan

**Affiliations:** College of Forestry, Sichuan Agricultural University, Sichuan Province Key Laboratory of Ecological Forestry Engineering on the Upper Reaches of the Yangtze River, State Forestry and Grassland Administration Key Laboratory of Forest Resources Conservation and Ecological Safety on the Upper Reaches of the Yangtze River, Chengdu, China

**Keywords:** *Cupressus funebris*, fine root, root system architecture, response strategy, soil environment

## Abstract

The root system architecture (RSA), being a key characteristic of the root economic spectrum, describes the spatial arrangement and positioning of roots that determines the plant's exploration of water and nutrients in the soil. Still, it remains poorly understood how the RSA of woody plants responds to the demand for water and nutrients in different soil environments and how the uptake of these resources is optimized. Here we selected single-species plantations of *Cupressus funebris* and determined their topological index (*TI*), revised topological index (*q*_*a*_ and *q*_*b*_), root link length (*RLL*), root branching rate (*R*_*b*_ and *R*_*i*_:*R*__*i*+_1_), and *in situ* soil physicochemical properties to assess which root foraging strategies adopt in different soil environments among Guang'an City (GA), Suining City (SN), Mianyang City (MY), and Deyang City (DY) in China. We also tested the potential effects of different nutrients upon RSA according to its plastic phenotype. Principal component analysis (PCA) showed that levels of soil nutrients were the highest at DY, followed by MY and SN, and lower at GA. A dichotomous branching pattern was observed for GA, SN, and MY, but a herringbone branching pattern for DY. The *RLL* was ranked as GA, > SN, > MY > DY. The *R*_*b*_ of GA, SN, and MY was significantly lower than that of DY (*p* < 0.05). Among the different city regions, values of *R*_1_*/R*_2_ were the largest in different regions and those of *R*_4_*/R*_5_ the smallest. The cross-sectional area of the root system did not differ between any two connected branch orders. The *TI, q*_*a*_, and *RLL* were significantly and negatively correlated with soil's water content, porosity, total nitrogen, total potassium, available nitrogen, and available phosphorus (*p* < 0.05), whereas they all had significant, positive relationships with soil temperature (*p* < 0.05). The *R*_*b*_ was significantly and positively correlated with total potassium in soil (*p* < 0.05). Redundancy analysis showed that total potassium was the main factor driving variation in RSA. Our results emphasize that the RSA is capable of corresponding plastic alterations by changing its number of internal or external links and the root link length of fine roots *vis-à-vis* a heterogeneous environment, thereby optimizing the rates of water capture and space utilization.

## Highlights

- The changing of root system architecture to different soil environments represents the strategy of adaptation and evolutionary direction of plants.
- The soil water and soil available potassium were the leading elements influencing the changes of *C. funebris* root system architecture.
- In order to adapt to the nutrient-poor soil environment, *C. funebris* would take a series of strategies, such as, increasing the number of internal links, the root link length, the root branching rate of low-orders fine roots, reducing the number of external links, and changing the root system architecture from a herringbone branching pattern to a dichotomous branching pattern.
- The cross-sectional area of the root system was basically equal to the sum of the cross-sectional areas of the root branches at any level in four test sites, which was in accordance with the Priciple of Leonardo's rule.

## Introduction

The root system architecture (RSA), as a pivotal characteristic of the root economic spectrum, refers to the complex physical connectivity belowground of plant parts (i.e., first-order, second-order, *i-*th-order, and primary roots), linking the root tips of different root branching, which serves as a networked channel for the circulation of plant matter, energy, and information (Karlova et al., 2021). Compared with the morphological characteristic of fine roots, the RSA is more important for the absorption and uptake of nutrients and water (Luo et al., 2020). At present, however, most knowledge of RSA has come from crops, such as *Oryza sativa* L. (De Bauw et al., 2019), *Zea mays* L. (Shao et al., 2018), and *Glycine max* (Linn.) Merr. (Durigon et al., 2019), in addition to *Arabidopsis thaliana* (Oláh et al., 2020). Hence, our understanding of RSA is still inadequate, especially for woody plants, the main constituents of forested terrestrial ecosystems, whose large and multiple root systems are hidden deeper underground, which increases the difficulty of exploring their structure and function (Zhou et al., 2019).

Previous studies of RSA have mainly relied on root growth or related characteristics, such as root diameter, specific root length, specific root surface area, and fine root biomass (Dorairaj et al., 2020; Madsen et al., 2020; Caruso et al., 2021). Moreover, these parameters cannot intuitively describe the spatial phenotypes of the distribution, arrangement, and location of fine roots in the soil, the growth medium (Luo et al., 2020). RSA's key parameters, namely, its topological index (*TI*), number of the root branches, root branching rate (*R*_*b*_), and root link length (*RLL*), are rarely studied, although they are recognized theoretically for being very important in plants' competition and allocation of resources. For instance, Wang et al. (2006) found that the *R*_*b*_ of the second-order roots was critical for roots' uptake of nutrients and water. Trubat et al. (2012) reported that fewer nitrogen and phosphorus elements reduced the topological index of a root system, which tended toward a herringbone branching pattern. Recently, Yildirim et al. (2018) pointed out that incorporating the lateral root link length can help to build a more efficient root system for water uptake in plants adapting to drought conditions. Yan et al. (2019) reported that nitrogen deposition tended to decrease the number of root branches in all root orders. Li et al. (2021) showed that a dichotomous branching pattern is beneficial in cold conditions. In general, there are still a few studies on RSA, and the results are still not unified.

The RSA is inevitably shaped by multiple stress factors in the environment, resulting in great differential plasticity in phenotype within and among plant species (Correa et al., 2019). For instance, Kawa et al. (2016) found that an inorganic phosphate deficiency shortened the main root length and increased the number of lateral roots. Work by Luo et al. (2020) emphasized that high temperature could significantly increase the branching intensity, though it decreased the average root diameter. According to Shao et al. (2018), higher competitive pressure can reduce the number of nodal roots, the density of lateral roots, and the root length of average lateral roots. In general, the RSA of plants would differentiate in response to a single stress. Nonetheless, the phenotypic plasticity of the RSA has yet to be empirically clarified in varied soil environments, mainly because of antagonistic, synergistic, or complementary effects among interacting environmental factors (Maurel and Nacry, 2020) and, in tandem, the heterogeneous demands of nutrients across space and time during the life history of plants (Giehl and von Wirén, 2014; Morris et al., 2017). Therefore, continuing to study the response strategy of RSA to different habitats is of great significance for exploring the adaptability and the survival strategies that woody plants must face under future climatic changes.

*Cupressus funebris* Endl is a common coniferous tree in the upper reaches of the Yangtze River, where it is significant for promoting soil and water conservation, combating desertification, and restoring vegetation (Wang et al., 2021). As a consequence of extensive logging and mismanagements, the growth and development of forest stands dominated by *C. funebris* are poor, rendering the forest ecosystem more fragile (Wu and Qi, 2021). In this context, most of the previous studies on ecological restoration have focused on stand structure (Baran et al., 2020), the diversity of understory species (Wang et al., 2021), and the soil physicochemical parameters, whereas few studies on root systems have also paid attention to their distribution, decomposition, and release (Chen et al., 2021), or the biomass and morphology of fine roots (Sierra Cornejo et al., 2020). Elucidating the mechanism underpinning the response and adaption strategy of RSA to habitat changes is therefore of great significance for optimizing forest ecosystem functioning.

In this study, we selected single-species *C. funebris* forests, analyzed the topological and branching structures of *C. funebris*'s RSA in different habitats, and then discussed the comprehensive effects of multiple factors in the soil environment upon this tree's fine RSA. The aim was to reveal the response mechanism and ecological adaptation strategies of RSA to habitat changes and to provide basic data and theoretical knowledge for an enhanced understanding of phenotypic plasticity mechanisms of the root systems.

## Materials and Methods

### Study Sites' Description

Based on the regional habitat conditions and their natural isolation, four ecological groups of single-species *C. funebris* plantations with large differences in their soil environment can be found distributed in the hilly area of central Sichuan, China. We selected four sampling sites, namely, Deyang City (DY), Suining City (SN), Mianyang City (MY), and Guang'an City (GA), representing the shallow hilly area, middle hilly area, deep (high) hill area, and low mountain area, respectively. These four sites lie within a subtropical monsoon climate zone, with stands of 25–30 years old.

We selected three standard plots (20 × 30 m) in each site, in May 2019, for a total of 12 (3 plots × 4 sites) established in this study. The following criteria were applied when selecting the location of each plot: less affected by human disturbances, far from the forest edge, with a canopy density at about 0.8 and similar vertical spatial. We surveyed and recorded the geographic information of each site, such as its latitude and longitude, altitude, aspect, and position; counted the number of woody plants; and measured tree height (with tree height measuring-instrument equipped with infrared distancing instrument) and diameter at breast height (DBH) with a tape (Table 1). In tandem, in the center of each plot, we buried (at a depth of 10 cm) a button thermometer (DS1921G), covered with a sealed bag and set to record every 2 h.

**Table 1:** General information of the four test sites used in the study.

**Index**	**Site**			
	**MY**	**DY**	**GA**	**SN**
Latitude	105°26′39″	104°25′41″	106°41′27″	105°31′57″
Longitude	31°15′54″	31°04′01″	30°04′58″	30°24′37″
Mean annual precipitation (mm)	880	1,150	1,150	930
Mean annual temperature (°C)	17.0	16.0	15.7	17.5
Elevation (m)	378	415	939	530
Average tree height (m)	7.5	9.3	6.5	8.4
Average diameter at breast height (DBH) (in cm)	11.5	12.6	8.8	12.1
Crown density	0.8	0.8	0.8	0.8
Slope gradient	25°	27°	28°	23°
Stand density (number·hm^−2^)	1,695	1,470	1,740	1,515
Slope aspect	Southeast	Southwest	Southeast	Southwest
Slope position	Under	Middle	Up	Up
Soil type	Alkaline soil	Weakly alkaline soil	Weakly acidic soil	Alkaline soil
Understory plants	*Smilax china*	*Myrsine africana*	*Myrsine africana*	*Myrsine africana*
	*Coriaria nepalensis*	*Adiantum capillusveneris*	*Coriaria nepalensis*	*Coriaria nepalensis*
	*Pogonatherum crinitum*	*Commelina communis*	*Smilax china*	*Ficus tikoua*
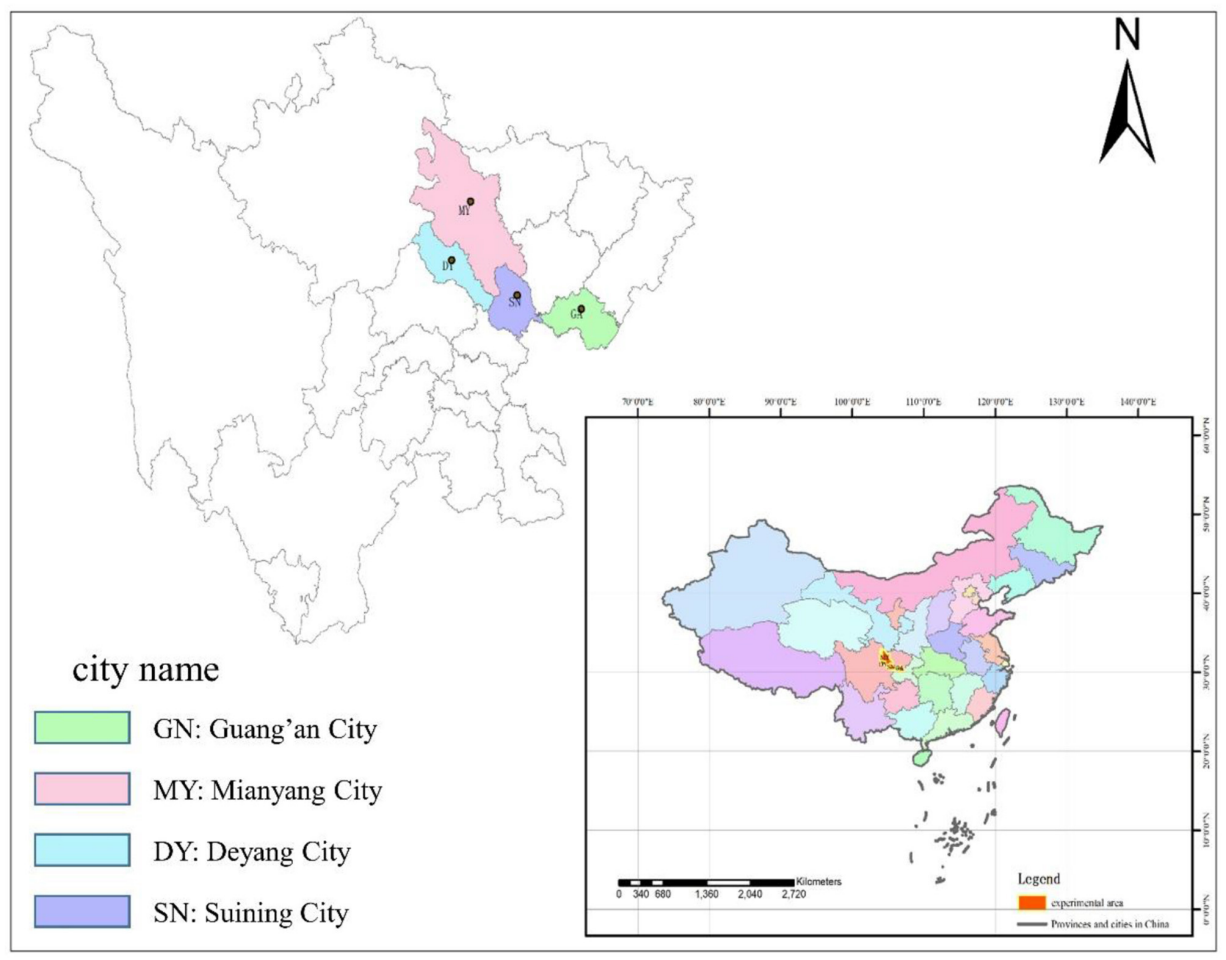				

### Experimental Design, Root Sampling, and Analysis

According to the average DBH and tree height, we selected three trees relatively scattered in each plot as standard trees, i.e., 36 trees (4 sites × 3 plots × 3 standard trees) per site, in September, 2020. Then, for each standard tree, we selected 1 or 2 of its woody main roots with integral 1- to 5-order fine roots, all in the same direction. If a woody main root was not obviously present along this direction, the main root within 5° of it on the left and right were chosen. The complete root system was then extracted using tools (shovel and brush) from the topsoil to the bottom soil and from the center to the periphery. At the same time, we carefully removed the soil attached to the surface root system. All the root and soil samples were stored in an icebox and taken to the laboratory. There, each was washed with running water, passed through a 0.15-mm mesh sieve, and stored in a fridge at low temperature (0–4°C).

The low temperature-stored roots were washed with deionized water at room temperature to remove any surface residues and then graded using the root order grading method (Pregitzer et al., 2002). Specifically, roots farthest from the main root axis of the root system, being root tips with no more branches, were defined as the 1-order fine root; the parent root of the 1-order fine root was defined as the 2-order fine root; and so on, until the 5-order fine root was reached. In addition, roots borne on a higher order fine root without branches were also classified as a 1-order fine root.

We recorded the number of internal and external links in the root system and used the Vernier caliper (accuracy: 0.01 mm), tape measure (accuracy: 1 mm), Epson digital scanner (Expression 10000XL 1.0), and root system-imaging analysis software (Win RHIZO Pro2009c) to measure and quantitatively analyze the diameter, internal and external links length, root length, and root diameter before or after root branching points.

### Soil Sampling, Physical, and Chemical Properties Analysis

First, soil temperature (ST) data were recorded with a button thermometer (DS1921G) when sampling the root system. Then, we sampled the *in situ* topsoil layer *ca*. 50 cm away from the trunk of each standard tree by using the ring-knife method to determine the soil bulk density (SBD) and soil porosity (SP). Finally, we also collected the surface topsoil *ca*. 0–50 cm away from the standard tree in the horizontal direction. After removing the litter and humus layer on the soil surface, we collected three soil samples with a 5-cm diameter drill at a depth of 0–20 cm. This depth was chosen since fine roots were predominantly restricted to the upper soil layers (Brassard et al., 2009) and simultaneously the relationships between fine roots, and soil physicochemical properties should be stronger compared to the deeper layers (Zema et al., 2021). After removing impurities (such as rocks, fine roots, and plant litter), all soil samples were mixed into one composite sample per tree. To determine the physical and chemical properties of the soil—including soil water content (SW), soil pH (pH), soil organic carbon (SOC), soil total nitrogen (STN), soil total phosphorus (STP), soil total potassium (STK), soil available nitrogen (SAN), soil available phosphorus (SAP), and soil available potassium (SAK)—about 2.0 kg of the *in situ* soil subsamples obtained by quartering were taken to the laboratory. The SW was determined by the drying method, the pH was determined by the glass electrode method (water-to-soil ratio is 1:2.5), SOC was determined by the potassium dichromate oxidation-external heating method, STN was determined by the kjeldahl method, STP was determined by the alkali fusion-molybdenum antimony colorimetric method, STK was determined by atomic absorption spectrophotometry method, SAN was determined by alkaline hydrolysis diffusion method, SAP was determined by sodium bicarbonate extraction-molybdenum-antimony colorimetric method, and SAK was leached and determined with neutral ammonium acetate extraction-atomic absorption spectrophotometry determination.

### Root Parameter Calculations

We calculated the topological index (*TI*, Equation 1) (Fitter, 1994), revised topological index (*q*_*a*_ and *q*_*b*_, Equations 2 and 3) (Oppelt et al., 2001), the cross-sectional area of the root branching (Equation 4) (van Noordwijk et al., 1994), and the root branching rate (*R*_*b*_ and *R*_*i*_*:R*_*i*+*I*_, Equation 5) (Wang et al., 2006), using the equations in Table 2.

**Table 2:** Equations for calculating the RSA (root system architecture).

**Number**	**Index**	**Equation**	**Where**	**Reference**
1	*TI*	*TI*= lg*A*/lg*M*		(Fitter, 1994)
2	*q_*a*_*	$qa=\frac{a-1-lb_{vo}}{vo-1-lb_{vo}}$	*b* = *P*_*e*_/*v*_*o*_ *lb*_*vo*_ = ln *v*_*o*_/ln 2	(Oppelt et al., 2001)
3	*q_*b*_*	$qb=\frac{b-1-lb_{vo}}{\left(v_{o}+1\right)/2-1/v_{o}-lb_{vo}}$	*b* = *P*_*e*_/*v*_*o*_ *lb*_*vo*_ = ln *v*_*o*_/ln 2	(Oppelt et al., 2001)
4	*d*	$d^{2}=\alpha \overset{n}{\underset{i=1}{\sum }}d_{i}^{2}$		(van Noordwijk et al., 1994)
5	*R_*b*_*, *R_*i*_:R_*i*+1_*	*R*_*i*_:*R*_*i*+1_ = lg*N*_*i*_:lg*N*_*i*+1_		(Wang et al., 2006)

Specifically, *TI* is the topological index of the root system (0.5–1); *q*_*a*_ and *q*_*b*_ are the revised topological index; *A* or *a* is the total number of links in the longest individual path in the root system, from its root basal link to an external link; *M* or *v*_*o*_ is the total number of external links that extend from a link in the root system that includes those ending with a meristem; *b* is the average topological length; *P*_*e*_ is the total links from all the base to the terminal; α is the ratio of the total root area before to after root branching; *d* is the diameter of the root system before branching; *d*_*i*_ is the diameter of the *i*-th root after root branching (*i* = 1, 2, 3, and 4); *R*_*b*_ (the total root branching rate) is represented by the inverse logarithm of the slope of the regression line; and *N*_*i*_ and *N*__*i*+_1_ denote the number of the *i*-th fine root (*i* = 1, 2, 3, and 4).

### Statistical Analysis

Excel was used to manage, sort, and calculate the data, after which SPSS 20.0 software (SPSS 20.0 for windows, SPSS Ins., Chicago, IL, USA) was used for statistical analyses.

First, the Shapiro-Wilk test and Levene's test were used to determine the normality and homogeneity of variance for each group of variables, respectively, and the Box-Cox method was applied as needed to transform the non-normal and uneven variables (through its base-10 logarithmic conversion), so they had normal distributions and a stable variance.

Second, a principal component analysis (PCA) was used to analyze soil physicochemical parameters—SW, ST, SBD, SP, pH, SOC, STN, STP, STK, SAN, SAP, and SAK—at the four test sites by the package “factoxtra” for R (version 4.1.1, R Core Team, 2021).

Third, the differences in RSA (i.e., topological index [*TI*], revised topological index [*q*_*a*_ and *q*_*b*_], *RLL* [root link length], root branching rate [*R*_*b*_ and *R*_*i*_:*R*__*i*+_1_]) and soil physicochemical parameters (i.e., SW, ST, SBD, SP, pH, SOC, TN, TP, TK, SAN, SAP, and SAK) among the four test sites were analyzed by one-way ANOVA using SPSS 20.0, followed by Duncan's multiple comparison method (*p* < 0.05). GraphPad Prism software (version 8.0.2) was used to draw the corresponding figures.

Fourth, Pearson correlations were performed between the RSA (*TI, q*_*a*_, *q*_*b*_, *RLL, R*_*b*_, and *R*_*i*_:*R*__*i*+_1_) and soil physicochemical parameters (SW, ST, SBD, SP, pH, SOC, TN, TP, TK, SAN, SAP, and SAK) in R (version 4.1.1) with the package “psych,” “pheatmap,” and “ggcorrplot.”

Finally, a redundancy analysis (RDA) was done to explore the relationships between the RSA (*TI, q*_*a*_, *q*_*b*_, *RLL, R*_*b*_, and *R*_*i*_:*R*__*i*+_1_) and soil physicochemical parameters (SW, ST, SBD, SP, pH, SOC, TN, TP, TK, SAN, SAP, and SAK); this was implemented in Canoco software (version 5.0).

## Results

### Soil Physicochemical Parameters in the Different City Sites

**Figure 1:**
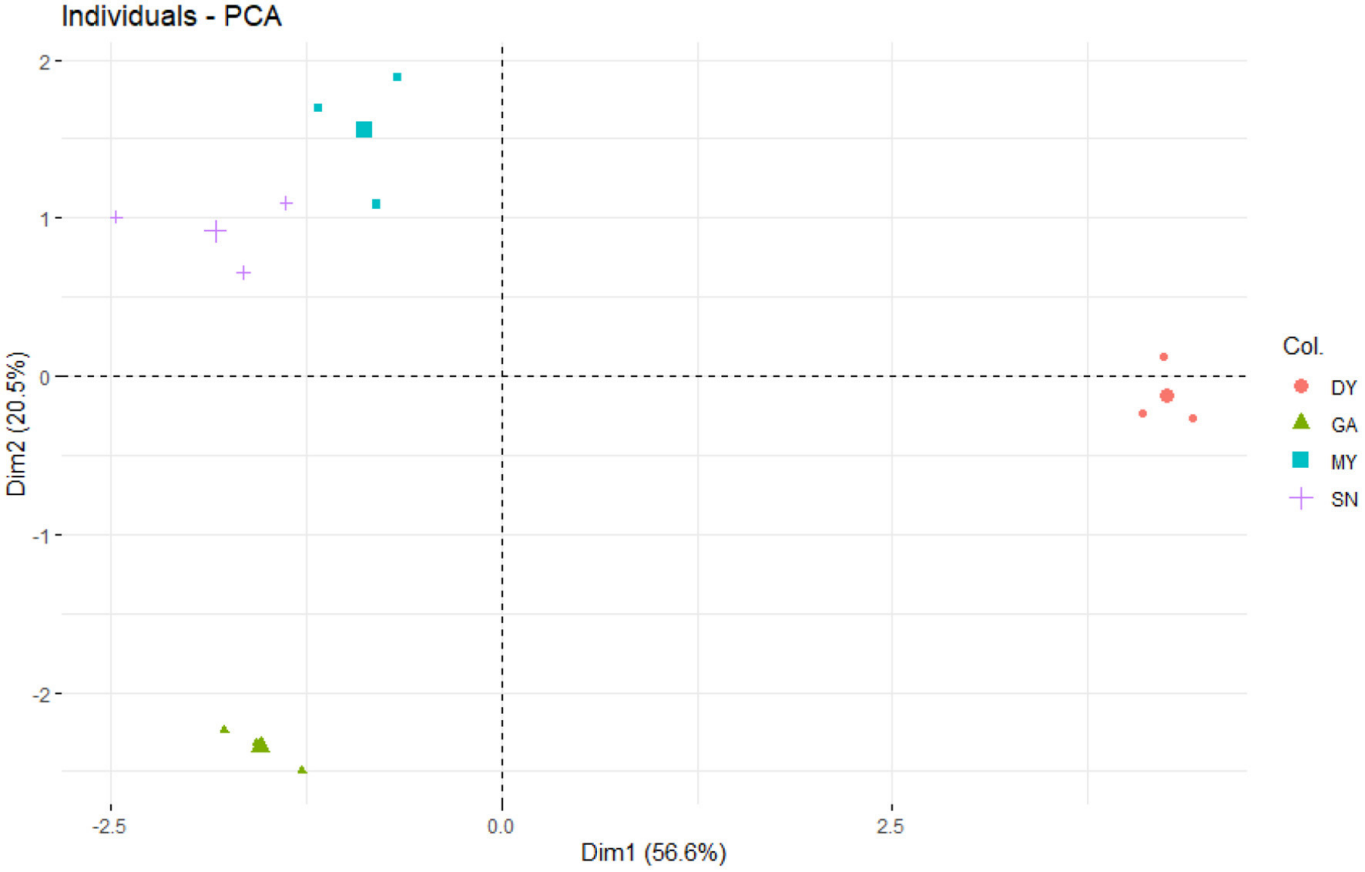
Principal component analysis (PCA) of the soil physicochemical parameters in the four sampled sites. DY, Deyang City; GA, Guang'an City; MY, Mianyang City; SN, Suining City.

The PCA (Figure 1) showed that most of the covariation among soil physicochemical parameters was represented by two independent dimensions. The first principal component (PC1) and second principal component (PC2) could be interpreted as the sources of variation for soil physicochemical parameters, explaining 56.6% and 20.5% of the total variation in the data. Their combined explanatory power for soil physicochemical parameters in four sampled regions was 77.1% (Figure 1). Overall, soil nutrients were the highest at DY, followed by MY and SN, and the lowest at GA. Specifically, the trends for SW, ST, SP, SOC, STN, STP, STK, SAN, SAP, and SAK were all similar, being ranked as follows: DY > MY > SN > GA, whereas the trends for soil pH (SN > MY > DY > GA) and SBD (MY > GA > SN > DY) differed and featured substantial within-treatment variation.

**Figure 2:**
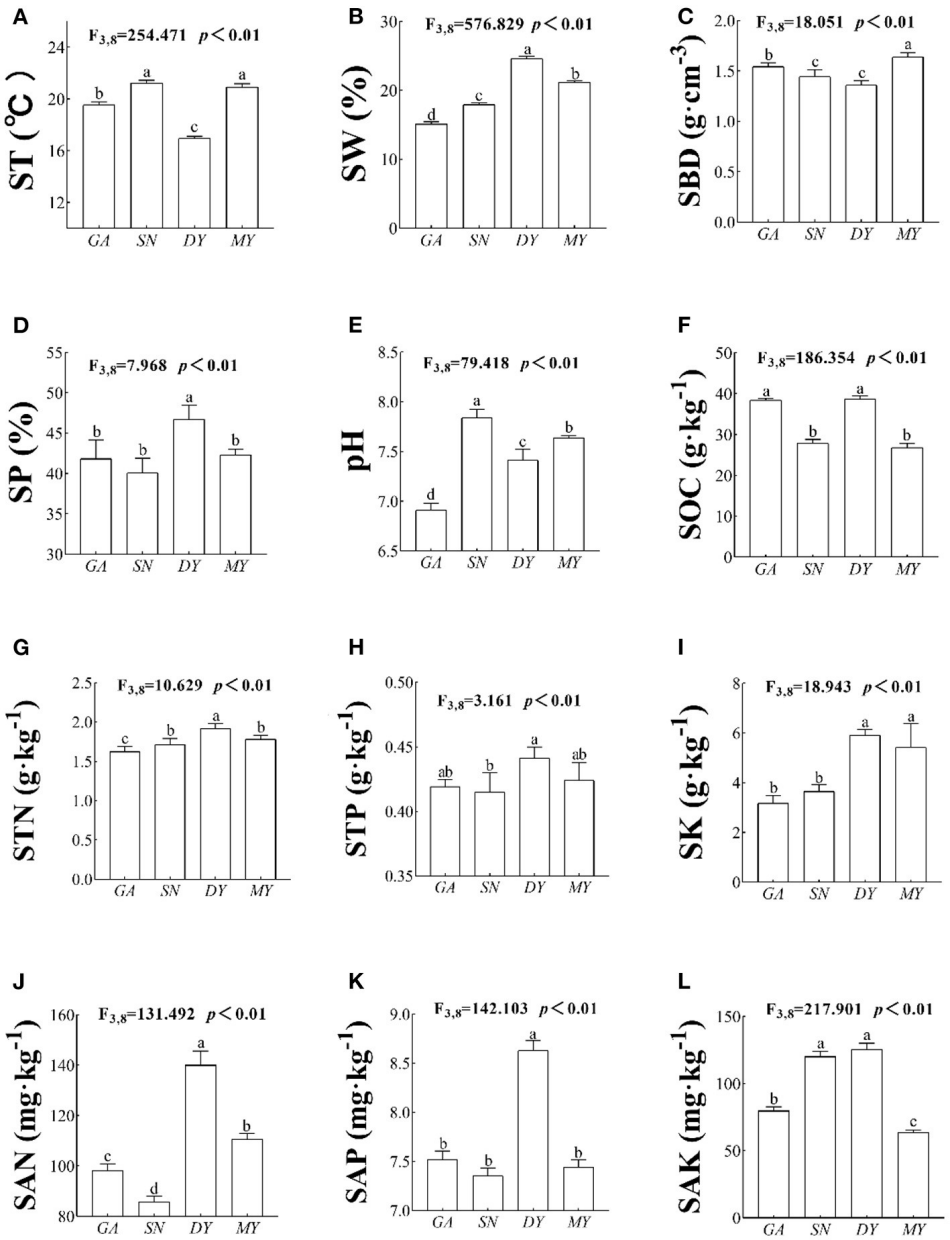
Soil physicochemical parameters in four sites. DY, Deyang City; GA, Guang'an City; MY, Mianyang City; SN, Suining City. The RSA index variables are **(A)** ST, soil temperature; **(B)** SW, soil water content; **(C)** SBD, soil bulk density; **(D)** SP, soil porosity; **(E)** pH, soil pH; **(F)** SOC, soil organic carbon; **(G)** STN, soil total nitrogen; **(H)** STP, soil total phosphorus; **(I)** SK, soil total potassium; **(J)** SAN, soil available nitrogen; **(K)** SAP, soil available phosphorus; and **(L)** SAK, soil available potassium. Data shown are the mean ± standard deviation (*n* = 3). Different lowercase letters indicate significant differences among the sites (*p* < 0.05).

There were significant differences in ST (*F*_3,8_ = 254.471, *p* < 0.01, Figure 2A), SW (*F*_3,8_ = 576.829, *p* < 0.01, Figure 2B), SBD (*F*_3,8_ = 18.051, *p* < 0.01, Figure 2C), SP (*F*_3,8_ = 7.968, *p* < 0.01, Figure 2D), pH (*F*_3,8_ = 79.418, *p* < 0.01, Figure 2E), SOC (*F*_3,8_ = 186.354, *p* < 0.01, Figure 2F), STN (*F*_3,8_ = 10.629, *p* < 0.01, Figure 2G), STP (*F*_3,8_ = 3.161, *p* < 0.01, Figure 2H), STK (*F*_3,8_ = 18.943, *p* < 0.01, Figure 2I), SAN (*F*_3,8_ = 131.492, *p* < 0.01, Figure 2J), SAP (*F*_3,8_ = 142.103, *p* < 0.01, Figure 2K), and SAK (*F*_3,8_ = 217.901, *p* < 0.01, Figure 2L) among the four sites. These results showed that the physical and chemical properties of the soil were not the same across the four sampled sites; hence, it would be promising to explore the response mechanism of RSA across this changed habitat and the associated ecological adaptation strategies.

### Root System Architecture of *C. funebris* in the Different City Regions

**Figure 3:**
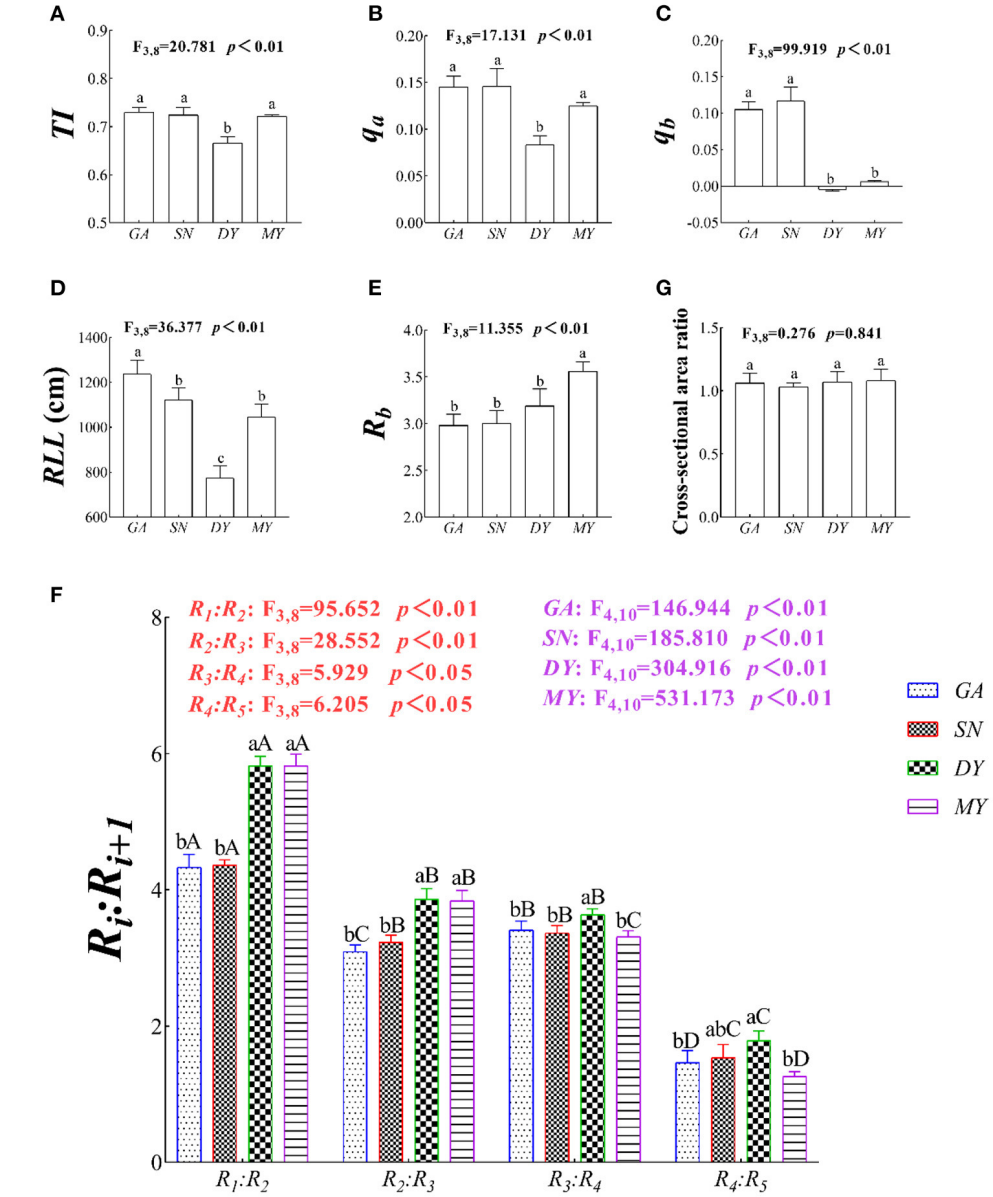
The root system architecture (RSA) at the four test sites. DY, Deyang City; GA, Guang'an City; MY, Mianyang City; SN, Suining City. The RSA index variables are **(A)** TI, topological index; **(B)** q_a_ and **(C)** q_b_, revised-topological index; **(D)** RLL, root link length; **(E)** R_b_, root branching rate; and **(F)** Ri:Ri+1, gradual root branching rate. Data shown are the mean ± standard deviation (n = 3). Different lowercase letters indicated significant differences among the sites (*p* < 0.05) in **(A–G)**. Different capital letters indicated significant differences among fine root-orders (*p* < 0.05) in **(F)**.

At the plot scale, the *TI, q*_*a*_, and *q*_*b*_ of *C. funebris* ranged from 0.67 to 0.73, 0.08 to 0.15, and 0.00 to 0.12, respectively. In other words, the root system of *C. funebris* was a transition type between a herringbone branching pattern and a dichotomous branching pattern. The *TI* (*p* < 0.01, *F*_3,8_ = 133.518, Figure 3A), *q*_*a*_ (*p* < 0.01, *F*_3,8_ = 82.68, Figure 3B), *q*_*b*_ (*p* < 0.01, *F*_3,8_ = 275.864, Figure 3C), and *RLL* (*p* < 0.05, *F*_3,8_ = 36.377, Figure 3D) of *C. funebris* root system differed significantly different across the four city regions. Specifically, a dichotomous branching pattern with more secondary branches was observed in GA, SN, and MY whereas a herringbone branching pattern in DY. *C. funebris* roots had increased the number of external links and reduced the root link length in nutrient-poor soils.

Across the different sites, the *R*_*b*_(*F*_3,8_ = 11.355, *p* < 0.05, Figure 3E), *R*_1_:*R*_2_ (*F*_3,8_ = 95.652, *p* < 0.05, Figure 3F), *R*_2_:*R*_3_ (*F*_3,8_ = 28.552, *p* < 0.05, Figure 3F), *R*_3_:*R*_4_ (*F*_3,8_ = 5.929, *p* < 0.05, Figure 3F), and *R*_4_:*R*_5_ (*F*_3,8_ = 6.205, *p* < 0.05, Figure 3F) of *C. funebris* ranged from 2.98 to 3.56, 4.33 to 4.82, 3.09 to 3.86, 3.31 to 3.64, and 1.26 to 1.79, respectively, with a large within-treatment variation. Notably, the *R*_*b*_ and *R*_*i*_:*R*__*i*+_1_ were significantly higher at DY than MY, SN or GA (*p* < 0.05, Figures 3E,F). That is, *C. funebris* trees had weakened internal competition, increased branching rate of lower order fine roots, and reduced branching rate of higher order fine roots, presumably in response to poor soil resources.

The root cross-sectional area ratio of GA, SN, MY, and DY were 1.06, 1.03, 1.08, and 1.07, respectively (Figure 3G), and not significantly different (*p* > 0.05). The cross-sectional area of the root system was basically equal to the sum of the cross-sectional areas of the root branches at any level in four test sites, thus following the principle of Leonardo's rule (Oppelt et al., 2001).

### Relationships Between RSA Index Variables and Soil Physicochemical Parameters

**Figure 4:**
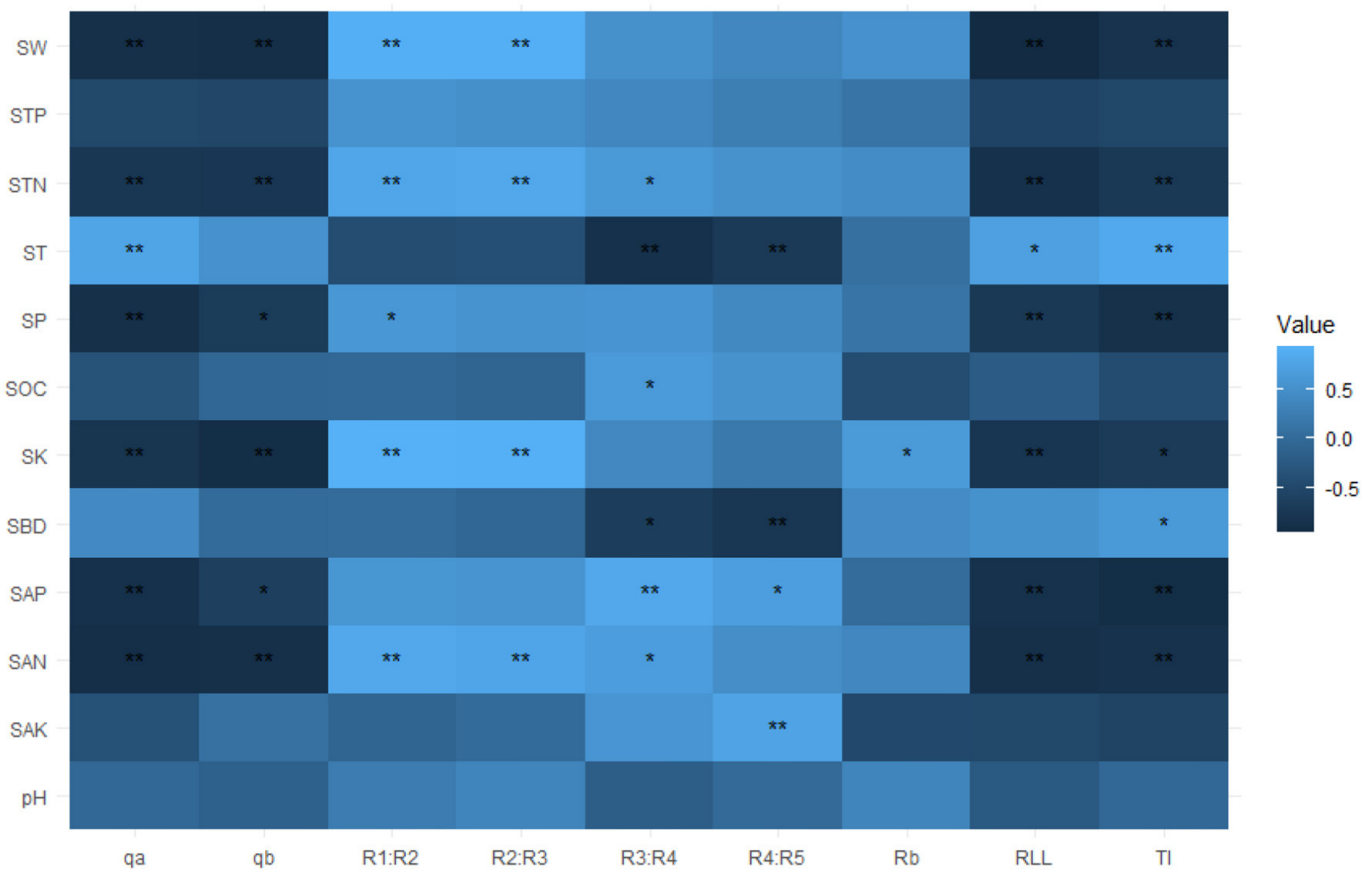
Correlation coefficients between RSA (root system architecture) and soil physicochemical parameters. The red and blue colors denote significant positive and negative effects, respectively. The color intensity is proportional to the magnitude of the Pearson correlation. The darker the color, the greater the strength of the relationship, and vice versa. The RSA index variables are TI, topological index; q_a_ and q_b_, revised topological index; RLL, root link length; R_b_, root branch rate; R_i_:R_i+1_, gradual root branch rate. The soil parameters are ST, soil temperature; SW, soil water content; SBD, soil bulk density; SP, soil porosity; pH, soil pH; SOC, soil organic carbon; STN, soil total nitrogen; STP, soil total phosphorus; SK, soil total potassium; SAN, soil available nitrogen; SAP, soil available phosphorus; SAK, soil available potassium. The * and ** indicate significance at *p* < 0.05 and *p* < 0.01, respectively.

The soil physicochemical parameters influenced the RSA index variables. First, we used Pearson correlations to analyze how these factors might be related to the RSA index variables. These results showed that the *q*_*a*_, *q*_*b*_, *TI*, and *RLL* were all significantly and negatively correlated with SW, SP, STN, SK, SAN, and SAP (*p* < 0.05, Figure 4), while *q*_*a*_, *TI*, and *RLL* were significantly and positively correlated with ST (*p* < 0.05, Figure 4). The *R*_*b*_ was significantly and positively correlated with SK (*p* < 0.05, Figure 4). Both *R*_1_:*R*_2_ and *R*_2_:*R*_3_ were significantly and positively correlated with SW, SP, STN, SK, and SAN (*p* < 0.05, Figure 4). The *R*_3_:*R*_4_ was significantly and positively correlated with STN, SOC, and SAN, yet vice versa with SBD and ST (*p* < 0.05, Figure 4). The *R*_4_:*R*_5_ had significant positive correlations with SAP and SAK, but negative correlations with SBD and ST (*p* < 0.05, Figure 4). To sum up, the RSA of *C. funebris* was characterized by heterogeneous phenotypic plasticity in the different soil environments.

**Figure 5:**
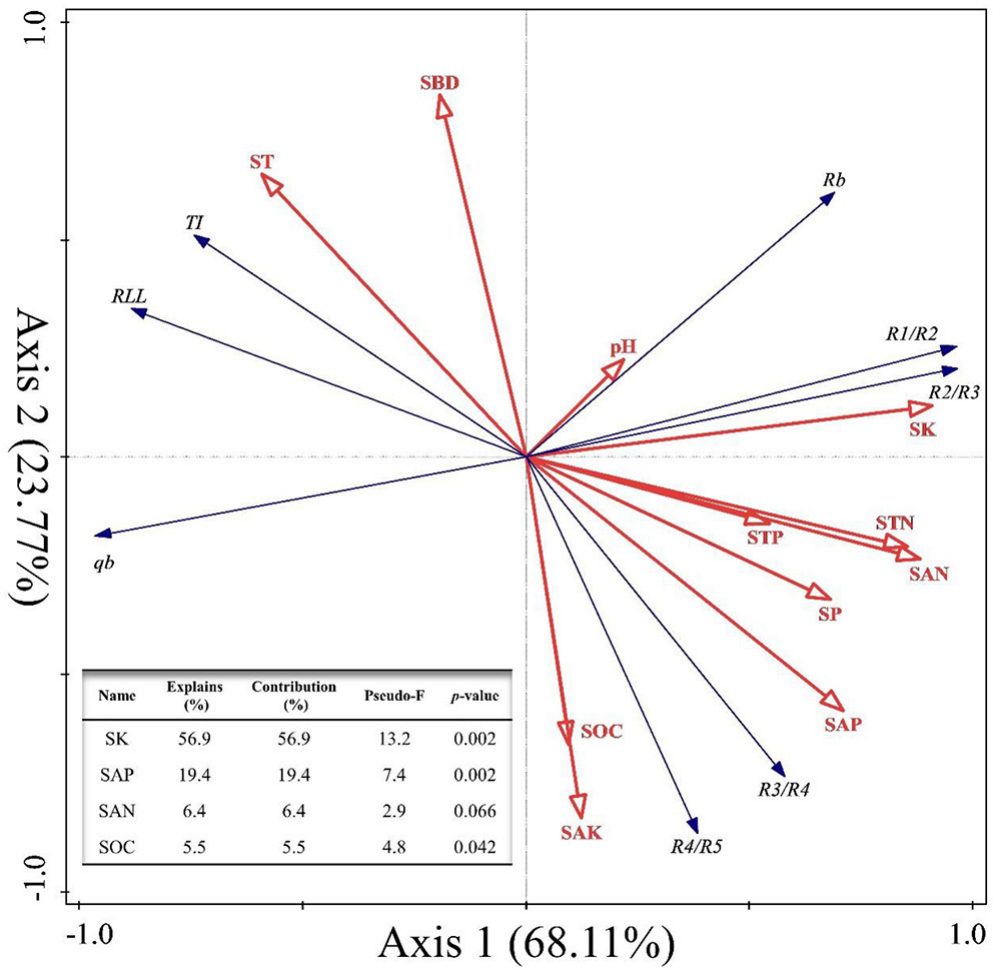
Redundancy analysis (RDA) of root system architecture (RSA) and soil physicochemical properties at the four different sites. RSA variables are TI, topological index; q_a_ and q_b_, revised-topological index; RLL, root link length; R_b_, root branching rate; R_i_:R_i+1_, gradual root branching rate. The soil parameters are SW, soil water content; SBD, soil bulk density; SP, soil porosity; pH, soil pH; SOC, soil organic carbon; STN, soil total nitrogen; STP, soil total phosphorus; SK, soil total potassium; SAN, soil available nitrogen; SAP, soil available phosphorus; SAK, soil available potassium. The angle between the two vector arrows can represent a significant and positive relationship (acute angle, <90°) or a negative relationship (obtuse angle, >90°). The length of each arrow is proportional to the magnitude of the standardized path coefficients (i.e., the strength of the relationship).

Second, we explored the effects of soil physicochemical parameters on the RSA index variables of *C. funebris* through an RDA (Figure 5). The interpretation of RDA's first axis and second axis amounted to 68.11% and 23.77%, respectively, for a combined contribution totaling 91.88% (Figure 5). Specifically, SK was the main factor driving variation in the RSA (*F* = 13.2, *p* = 0.002 <0.01, Figure 5), for which the explanatory and contributing percentages were 56.9% and 56.9%, respectively. Furthermore, the corresponding percentages for SAP were 19.4% and 19.4%, respectively (*F* = 7.4, *p* = 0.002 <0.01, Figure 5). Accordingly, the joint contribution by these two factors surpassed that of all other soil physicochemical parameters combined, distinguishing the two as the main and significant factors affecting the RSA (*p* < 0.05).

## Discussion

This study investigated heterogeneity in soil conditions as they affect RSA of a woody plant. The *TI* indirectly describes the uptake efficiency of roots for water and nutrients, for which Fitter (1994) proposed two extreme branching patterns, namely, the dichotomous branching pattern (*TI* = 1) to adapt to nutrient-poor soils and the herringbone branching pattern for the nutrient-rich soils (*TI* = 0.5). However, due to the influences from plant growth characteristics, soil fertilities, and soil compaction, the *TI* values always range from 0.5 to 1 (Fitter, 1994; Magalhães and Seifert, 2015; Li et al., 2020, 2021; Hao et al., 2021). In our study, the root system of *C. funebris* branching pattern changed from a herringbone branching pattern (having a small number of internal links, many external links, many secondary branches, and internal overlaps) to a dichotomous branching pattern exhibiting more internal links, fewer external links, fewer secondary branches, less overlap, and weaker internal competition (Figures 3A–C). Searching the literature, we found several studies similar to ours. For instance, Trubat et al. (2012) reported that with lower nitrogen and phosphorus, the root system topological index was reduced and tended toward a herringbone branching pattern. Later, Correa et al. (2019) summarized that soil compaction was significantly and positively correlated with the topological index; i.e., the dichotomous branching pattern of RSA was a way for plants to adapt to the higher strength of soil compaction. Li et al. (2020) argued that root systems shifted from a herringbone branching pattern to a dichotomous branching pattern when plants adjust to drought stress. More recently, Li et al. (2021) also showed that a dichotomous branching pattern is beneficial to cold resistance. Yet, work by Hao et al. (2021) failed to find soil texture influencing the topological index. In particular, compared with Hao et al.'s (2021) experiment, their extracted SBD and SP values are similar to those in our study, and we found that soil texture was significantly and positively correlated with the topological index. In general, plants' survival strategies lean toward increasing the number of internal links or reducing the number of external links when adapting to the soil nutrient-poor soil environments.

Increasing the root link length of the root system is a vital strategy for plants to improve the distribution range of their roots in the soil and optimize the uptake of these resources (Yildirim et al., 2018). The *RLL* clearly differed geographically (Figure 3D), which suggests that *C. funebris* trees could pursue an expansion strategy whereby root link length is altered to adapt to local environmental changes. The root link length conveys the distribution range of roots and the expansion ability of the root system in soil (Wu et al., 2016). It followed that increasing the root link length could expand the distribution range of the root system in the belowground soil space. For example, Yildirim et al. (2018) found that a larger lateral root link length promoted water uptake to adapt to drought. Yan et al. (2019) pointed out that the addition of nitrogen to soil could increase the root link length of the lower order fine roots through their faster growth rate. Fundamentally, in order to phenotypically adapt to a nutrient-poor soil environment, the roots of *C. funebris* would adopt a survival strategy of increasing its root link length.

The branching rate is a very sensitive parameter of RSA, one that can reflect the plasticity of root systems to different site conditions, forest stand ages, or stages of forest succession (Duque and Villordon, 2019). In our study, the average branching rate was 3.18 (varying from 2.98 to 3.56), and the root system's *R*_1_*/R*_2_ was the highest, while its *R*_4_*/R*_5_ was the lowest (Figures 3E,F). These results are in line with many previous reports. For instance, Wang et al. (2006) reported a root branching rate of woody plants spanning 3.0–6.0, which decreased when the higher order of fine roots increased. Similarly, Liese et al. (2017) also showed that the root branching rate of woody plants ranged from 2.2 to 3.0, whose *R*_*b*_ affected the roots' chemical composition (e.g., root carbon:nitrogen ratio) through certain root morphological traits (e.g., the specific root length). In addition, the branching rates of *C. funebris* were significantly heterogeneous to adapt to different soil conditions in this study, similar to many other findings. Bécel et al. (2012) demonstrated that the root branching rate decreased significantly to enable plants to adapt to greater SBD. Li et al. (2017) found that precipitation during the growing season increased the branching rate, especially, increasing the higher order fine roots more so than the first-order fine roots. Meier et al. (2020) showed that available nitrogen promoted the lateral root formation and development to build a higher ordered root branched system. Finally, as noted by Motte and Beeckman (2019), increasing the ability of root branching was conducive to exploring the soil for water and nutrients. Collectively, *C. funebris* tended to enhance its absorption efficiency by increasing the root branching of its lower order fine roots to adapt to nutrient-poor regions.

Soil is in direct contact surface with the root system, and is it incontrovertible that the growth and development of plant root systems are affected by one or more soil physicochemical properties (Correa et al., 2019; Wu et al., 2021). However, *C. funebris* is a non-mycorrhizal tree species (Wang et al., 2021); hence, it relies on absorbance by its lateral root system to obtain mineral elements, such as nitrogen, phosphorus, and potassium, to maintain its growth (Trubat et al., 2012; Kiba and Krapp, 2016). In our study, STK exerted the most pronounced effect upon RSA (Figures 4, 5), as seen in many studies with other woody plants (Julkowska et al., 2017; Mai et al., 2019; Templalexis et al., 2021). There might be two reasons for this: (1) Potassium is a key element in the formation of lateral roots, whose accumulation is also beneficial to resist salt stress (Julkowska et al., 2017). (2) Potassium in the root system generates an osmotic pressure gradient between the root and soil, letting water enter the root system more easily from the soil to aboveground parts (leaves) for transport along the concentration gradient, to take part in photosynthesis and respiration processes and plays a regulatory role in downward transportation of photosynthetic products (Mai et al., 2019). Furthermore, the content and distribution of phosphorus would likely impact the RSA of fine roots in the soil (Figures 4, 5). For example, Yuan et al. (2016) argued that plants undertake a series of strategies such as increasing the root tip number and extending their root link length to counter a deficiency in available phosphorus.

Many studies had also pointed out that temperature would change the RSA of plants. For example, Qin et al. (2007) noted that a high temperature would inhibit both the root branching rate and root diameter. Later, Nagel et al. (2009) uncovered positive correlations for the root coefficient, root branching rate, root length, and root density versus temperature. Clark et al. (2011) proposed that temperature would change the RSA, in terms of its root length and root width. Luo et al. (2020) emphasized that temperature could adjust the strength and direction of RSA and is thereby presumably capable of also modulating the competitive relationship among plants. Nevertheless, in our research, although the temperature at four test sites did not differ significantly, the correlations between RSA index and temperature were very weak (Figures 4, 5). The differences in soil temperature among the four sites (Figure 2A) were all within the suitable region (16–22°C) for most tree species; hence, our results did not invalidate the conclusion of RSA remaining stable in a suitable temperature range. Additionally, Gray and Brady (2016) suggested that the responses of plants to increasing temperature could be species-specific due to conserved traits among species, given that the intraspecific optimal temperature varied litter, implying an evolutionarily conserved strategy. Furthermore, heterogeneity in temperature could also be offset or compensated by differential levels of nitrogen, phosphorus, potassium, and/or other nutrient elements, which could not only conform to the law of minimum factor but would also be in line with the law of complementarity among factors (Maurel and Nacry, 2020). Besides, some reports indicated that soil compaction would lead to thicker roots and higher tortuosity to strengthen the overall exploration ability of the root system (Tracy et al., 2012; Colombi et al., 2018; Correa et al., 2019). But, our study's results did not find evidence for that (Figures 2C,D). We suggest that soil compaction at the four sampled plantation sites was insufficient to induce significant changes in the RSA of *C. funebris*. That being said, the synergy or antagonism of multiple soil physicochemical parameters likely could have mitigated any negative effects caused by soil compaction (Maurel and Nacry, 2020).

## Conclusion

The changed RSA under different soil environments reveals the adaptation strategy and evolutionary direction of plants. In order to adjust to a nutrient-poor soil environment, our research results emphasize that the root system of *C. funebris* would employ a suite of strategies, such as increasing the number of internal links, the root link length, the root branching rate of low-order fine roots, reducing the number of external links, and changing the RSA from a herringbone branching pattern to a dichotomous branching pattern. Soil potassium was the main factor affecting RSA. In summary, our results highlight how RSA can demonstrate plastic changes in response to heterogeneity in the soil environment, which contributes to a better understanding of the response strategies to differing soil factors.

## Data Availability Statement

The original contributions presented in the study are included in the article/supplementary material, further inquiries can be directed to the corresponding author/s.

## Author Contributions

WH, CL, and CF designed the study. CL, YaW, YuW, XW, and TL collected the data and performed the analysis. WH and CL led the writing. GC, KZ, XL, and CF contributed to the revision. All authors contributed to discussion and writing, read, and approved the final manuscript.

## Funding

This study was supported by the German Government loans for Sichuan Forestry Sustainable Management (Grant No. G1403083) and the Key Sci-Tech Project of the 12th 5-Year Plan of China (Grant No. 2011BAC09B05).

## Conflict of Interest

The authors declare that the research was conducted in the absence of any commercial or financial relationships that could be construed as a potential conflict of interest.

## Publisher's Note

All claims expressed in this article are solely those of the authors and do not necessarily represent those of their affiliated organizations, or those of the publisher, the editors and the reviewers. Any product that may be evaluated in this article, or claim that may be made by its manufacturer, is not guaranteed or endorsed by the publisher.
